# Optimal allocation to treatment sequences in individually randomized stepped-wedge designs with attrition

**DOI:** 10.1177/17407745231154260

**Published:** 2023-02-24

**Authors:** Mirjam Moerbeek

**Affiliations:** Department of Methodology and Statistics, Utrecht University, Utrecht, The Netherlands

**Keywords:** Staggered intervention, optimal allocation, stepped-wedge trial, constrained optimization

## Abstract

**Background/aims:**

The stepped-wedge design has been extensively studied in the setting of the cluster randomized trial, but less so for the individually randomized trial. This article derives the optimal allocation of individuals to treatment sequences. The focus is on designs where all individuals start in the control condition and at the beginning of each time period some of them cross over to the intervention, so that at the end of the trial all of them receive the intervention.

**Methods:**

The statistical model that takes into account the nesting of repeated measurements within subjects is presented. It is also shown how possible attrition is taken into account. The effect of the intervention is assumed to be sustained so that it does not change after the treatment switch. An exponential decay correlation structure is assumed, implying that the correlation between any two time point decreases with the time lag. Matrix algebra is used to derive the relation between the allocation of units to treatment sequences and the variance of the treatment effect estimator. The optimal allocation is the one that results in smallest variance.

**Results:**

Results are presented for three to six treatment sequences. It is shown that the optimal allocation highly depends on the correlation parameter ρ and attrition rate r between any two adjacent time points. The uniform allocation, where each treatment sequence has the same number of individuals, is often not the most efficient. For 0.1≤ρ≤0.9 and r=0,0.05,0.2 , its efficiency relative to the optimal allocation is at least 0.8. It is furthermore shown how a constrained optimal allocation can be derived in case the optimal allocation is not feasible from a practical point of view.

**Conclusion:**

This article provides the methodology for designing individually randomized stepped-wedge designs, taking into account the possibility of attrition. As such it helps researchers to plan their trial in an efficient way. To use the methodology, prior estimates of the degree of attrition and intraclass correlation coefficient are needed. It is advocated that researchers clearly report the estimates of these quantities to help facilitate planning future trials.

## Introduction

**Figure 1.**
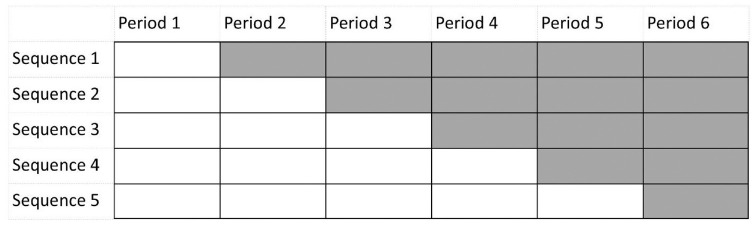
Graphical representation of a stepped-wedge design with five sequences.

Since the study by Hussey and Hughes,^1^ the stepped-wedge design has gained increasing attention in the medical statistical literature. The stepped-wedge design is a special type of the cross-over design^2,3^ in which cross-over only occurs from the control to the intervention condition. This is illustrated in Figure 1, in which five treatment sequences can be distinguished. In this figure, all sequences start in the control condition and at the beginning of each time period one sequence crosses over to the intervention. As both treatment conditions are available within each sequence, the design is more efficient than the multi-period parallel-group design with the same number of time periods. Furthermore, it may result in easier recruitment because everyone will eventually receive the intervention condition.

The implementation of a stepped-wedge design has seen an increasing use in cluster randomized trials.^4,5^ With such designs, complete clusters, such as households, family practices or clinics are randomized to treatment sequences. Of course, it is also possible to implement the stepped-wedge design in an individually randomized trial.^6^ Examples are trials to treat obstructive sleep apnoea,^7^ chronic constipation,^8^ malformations,^9,10^ dementia,^11^ to reduce household air pollution^12^ and to evaluate self-management support programmes^13^ and spillover of HIV knowledge.^14^ The design of the individually randomized stepped-wedge design has not yet been thoroughly explored in the statistical literature. A recent study evaluated the efficiency of the individually randomized stepped-wedge design in trials with three time periods.^15^

Stepped-wedge designs are often implemented such that an equal number of clusters or individuals is assigned to each treatment sequence (i.e. a uniform allocation). However, for cluster randomized trials, it has already been shown that this is not necessarily the best choice.^16–18^ It is, therefore, expected that a uniform allocation is not the best choice in an individually randomized stepped-wedge design either. The aim of this contribution is to study the optimal allocation of individuals to treatment sequences and the relative efficiency of the uniform allocation as compared to the optimal allocation. Furthermore, to what extent the optimal allocation changes if the study is hampered by attrition of individuals over time will also be studied.

This contribution is organized as follows. In the ‘Methods’ section, the statistical model that relates outcome to time period and treatment condition is introduced and it is shown how the treatment effect and its variance are estimated in studies without and with attrition. The variance of the treatment effect estimate is used as optimality criterion and this section also shows how constrained optimization is used to numerically derive the optimal allocation to treatment sequences. The ‘Results’ section presents optimal allocations for three to six sequences along with the efficiency of the uniform allocation relative to the optimal allocation. The optimal allocations may not always be feasible from a practical point of view and the ‘Methods’ section deals with optimal allocations where the proportions of individuals allocated to each sequence are bounded by an upper and lower limit. Conclusion and discussion are given in the last section.

## Methods

### Statistical model

All individuals start in the control condition, and in each time period a number of individuals crosses over the intervention. The number of time periods *T* in a stepped-wedge design as depicted in Figure 1 is T=J+1 , where J is the number of sequences. A measurement is taken at the end of each time period. The model for the quantitative outcome $Y_{\mathrm{ijt}}$ of individual $i=1,\;\ldots ,\;n_{j}$ in sequence j=1,…,J at the end of time period t=1,…,T is as follows

$$Y_{ijt}=\beta +\tau_{t}+x_{jt}\gamma +\varepsilon_{ijt}$$

Here, β is the baseline score, $\tau_{t}$ are the period effects (with $\tau_{1}=0$ for identifiability), $x_{\mathrm{jt}}$ is treatment condition (with $x_{\mathrm{jt}}=0$ if sequence j is in the control condition in time period t and 1 if it is in the intervention condition in this time period) and γ is the effect of treatment. Note that this treatment effect does not depend on the time elapsed since crossing over, hence it is a sustained effect. The residual is denoted as $\varepsilon_{\mathrm{ijt}}$ , and has a mean equal to zero and a variance that is equal to $\sigma_{\varepsilon }^{2}$ . The correlation between $\varepsilon_{\mathrm{ijt}}$ and $\varepsilon_{\mathrm{ij}t\prime }$ depends on the time difference between the measurements: $\mathrm{cor}(\varepsilon_{\mathrm{ijt}},\;\varepsilon_{\mathrm{ij}t\prime })=\rho^{\left|t-t\prime \right|}$ , where the parameter 0<ρ<1 denotes the correlation between two measurements one time period apart. This correlation function is called the exponential decay function:^19,20^ the correlation between two measurements becomes smaller if these two measurements are further away in time. Such a correlation function is more realistic than compound symmetry, where the correlation does not depend on the time lag between any two measurements.

The model can be written in matrix–vector notation. The model for individual $i=1,\ldots ,n_{j}$ in sequence j=1,…,J is given by

$$Y_{ij}=X_{j}\theta +\varepsilon_{jt}$$

where

$$Y_{\mathrm{ij}}=\left(\begin{array}{ccccc} Y_{\mathrm{ij}1} & Y_{\mathrm{ij}2} & Y_{\mathrm{ij}3} & \cdots & Y_{\mathrm{ijT}} \end{array}\right)\prime$$

is the vector of length T with responses

$$X_{j}=\left(\begin{array}{ccccc} 1 & 0 & 0 & \cdots & x_{j1}\\ 1 & 1 & 0 & \cdots & x_{j2}\\ 1 & 0 & 1 & \cdots & x_{j3}\\ \vdots & \vdots & \vdots & \ddots & \vdots \\ 1 & 0 & 0 & 1 & x_{\mathrm{jT}} \end{array}\right)$$

is the T×(T+1) matrix with predictors

$$\theta =\left(\begin{array}{cccccc} \beta & \tau_{1} & \tau_{2} & \cdots & \tau_{T} & \gamma \end{array}\right)\prime$$

is the vector of length T+1 with regression coefficients

$$\varepsilon_{\mathrm{ij}}=\left(\begin{array}{ccccc} \varepsilon_{\mathrm{ij}1} & \varepsilon_{\mathrm{ij}2} & \varepsilon_{\mathrm{ij}3} & \cdots & \varepsilon_{\mathrm{ijT}} \end{array}\right)\prime$$

is the vector of length T with residuals, and

$$V=\sigma_{\varepsilon }^{2}\left(\begin{array}{ccccc} 1 & \rho & \rho^{2} & \vdots & \rho^{T}\\ \rho & 1 & \rho & \rho^{2} & \vdots \\ \rho^{2} & \rho & 1 & \rho & \rho^{2}\\ \vdots & \rho^{2} & \rho & 1 & \rho \\ \rho^{T} & \vdots & \rho^{2} & \rho & 1 \end{array}\right)$$

is the T×T variance–covariance matrix of this vector. Note that each individual has the same matrix V , meaning that the parameters $\sigma_{\varepsilon }^{2}$ and ρ are constant across individuals.

Given an estimate $\hat{V}$ of the matrix V , the vector of regression coefficients is estimated as

$$\hat{\theta }={\left(\sum_{j=1}^{J}n_{j}X_{j}\prime {\hat{V}}^{-1}X_{j}\right)}^{-1}\sum_{j=1}^{J}n_{j}X_{j}\prime {\hat{V}}^{-1}y_{\mathrm{ij}}$$

with corresponding variance–covariance matrix

$$\mathrm{cov}\left(\hat{\theta }\right)={\left(\sum_{j=1}^{J}n_{j}X_{j}\prime {\hat{V}}^{-1}X_{j}\right)}^{-1}$$

The treatment effect estimate $\hat{\gamma }$ is the last entry of vector $\hat{\theta }$ ; its associated variance $\mathrm{var}(\hat{\gamma })$ is in row T and column T of matrix $\mathrm{cov}(\hat{\theta })$ .

The stepped-wedge design is a multi-period design, and it is very likely individuals drop out during the course of the study. The last observation of individual i in sequence j is taken at the end of time period $T_{\mathrm{ij}}$ , with $1\leq T_{\mathrm{ij}}\leq T$ . The number of individuals in sequence *j* who have their last observation at the end of time period t is denoted by $n_{\mathrm{jt}}$ . In the case of a constant attrition rate r between any two adjacent time points, $n_{\mathrm{jt}}=n_{j}(1-r{)}^{t-1}-n_{j}(1-r{)}^{t}$ . The matrix $X_{\mathrm{jt}}$ includes the first t rows of matrix $X_{j}$ and the matrix $V_{t}$ includes the first t rows and first t columns of matrix V . The variance–covariance matrix of the estimated regression coefficients then becomes

$$\mathrm{cov}\left(\hat{\theta }\right)={\left(\sum_{j=1}^{J}\sum_{t=1}^{T}n_{\mathrm{jt}}X_{\mathrm{jt}}\prime {{\hat{V}}_{t}}^{-1}X_{\mathrm{jt}}\right)}^{-1}$$

The proportion individuals allocated to sequence j is denoted $p_{j}$ , with inequality constraint $0<p_{j}<1,\forall j$ and equality constraint $\sum_{j=1}^{J}p_{j}=1$ . The optimal allocation of individuals to sequences is denoted $p^{*}=(p_{1}^{*},p_{2}^{*},\ldots ,p_{J}^{*})$ and minimizes the variance of the treatment effect estimator $\mathrm{var}(\hat{\alpha })$ . As such, the optimal allocation results in a treatment effect that is estimated with highest precision and hence power of the test on treatment effect is maximized.

In practical settings, the lower and upper bounds 0 and 1 for $p_{j}$ may result in an optimal allocation $p^{*}$ that is not feasible. For instance, it may be difficult to implement the intervention when the number of subjects in a sequence is either too low or too high. The inequality constraint $0<p_{j}<1$ may then be replaced by the set of constraints $p_{L_{j}}<p_{j}<p_{U_{j}}$ , where $p_{L_{j}}$ and $p_{U_{j}}$ are the user-specified lower and upper boundaries for sequence j . These boundaries have a subscript j , meaning that they may be different across the treatment sequences. They should be chosen such that the equality constraint $\sum_{j=1}^{J}p_{j}=1$ can still be met. The optimal allocation then becomes a constrained optimal allocation. The second subsection of the ‘Results’ section shows an example where constrained optimization is applied.

A simple equation for the relation between the allocation $p=(p_{1},p_{2},\ldots ,p_{J})$ and the variance $\mathrm{var}(\hat{\gamma })$ cannot be derived in the case of exponential decay and/or attrition. For that reason, the derivation of the optimal allocation to treatment conditions is done numerically, using the function constrOptim.nl in the R package Alabama^21^ for optimization with equality and inequality constraints. This function is iterative and requires a starting vector of proportions. It is advised to use various such vectors to avoid convergence to a local minimum of $\mathrm{var}(\hat{\gamma })$ . The function does not only report the optimal allocation, but also the value of $\mathrm{var}(\hat{\gamma })$ achieved for the optimal allocation. As such, the efficiency of the optimal allocation can be compared to any other allocation (Supplemental material).

### Relative efficiency

Once the optimal allocation has been derived, it can be compared to the uniform allocation. The relative efficiency quantifies the loss of efficiency of using the uniform allocation $p_{u}$ rather than the optimal allocation $p^{*}$ . It is calculated as $\mathrm{RE}=\;\mathrm{var}(\hat{\gamma }{)}_{p=p^{*}}/\;\mathrm{var}(\gamma {)}_{p=p_{u}}$ , where the numerator and denominator are the variances of the treatment effect estimator as obtained with the optimal and uniform allocation, respectively. The relative efficiency is in the interval [0,1] . A relative efficiency of 0.8 implies the sample size of the uniform allocation has to be increased by $( ( 1/0.8) - 1) \times 100{\rm%} = 25{\rm%}$ to perform as well as the optimal allocation; for a relative efficiency of 0.9, the sample size has to be increased by $( ( 1/0.9) - 1) \times 100{\rm%} = 11{\rm%}$.

## Results

### Optimal allocation to treatment sequences

**Figure 2.**
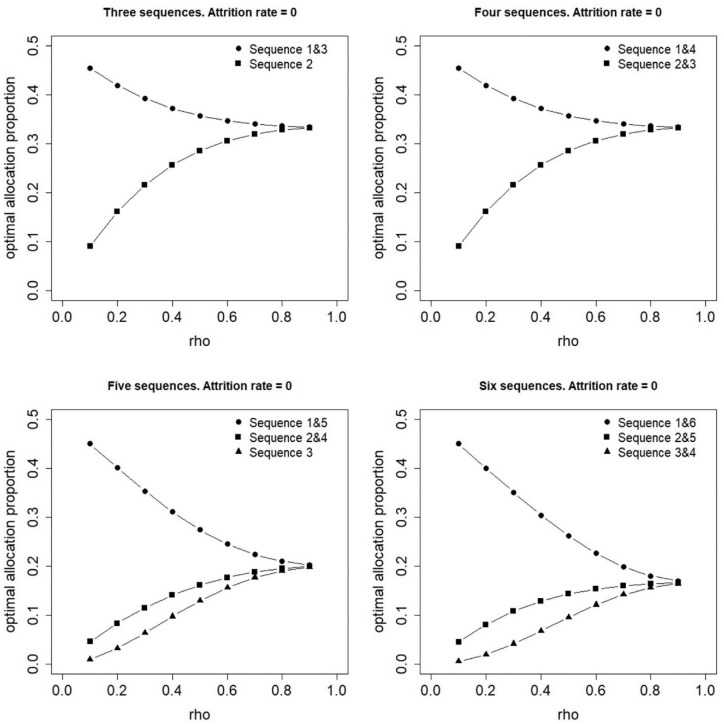
Optimal allocation of individuals to three, four, five or six treatment sequences in case attrition is absent.

Figure 2 shows the optimal allocation to treatment sequences as a function of the correlation parameter ρ and for three, four, five and six sequences in case attrition is absent (i.e. when r=0 ). The optimal allocation to sequences strongly depends on ρ : for small ρ , there is a large variability across the optimal proportions, $p_{j}$ , while the optimal allocation approaches the uniform allocation if ρ increases to 0.9. The optimal allocation holds symmetry properties: the first and the last sequence have equal optimal proportions, the second and the second-last sequence have equal optimal proportions, and so forth. Furthermore, the first and last sequence have highest optimal proportions, and the further away a sequence is from the top or bottom edges of the design (i.e. the closer the treatment switch is to the middle time period(s)), the lower the optimal proportion. A related result was previously found for the cluster randomized stepped-wedge design: the information content is higher for sequences closer to the edges.^22^ As is obvious, the higher the information content of a sequence, the more advantageous it is to assign a large proportion of individuals to that sequence.

**Figure 3.**
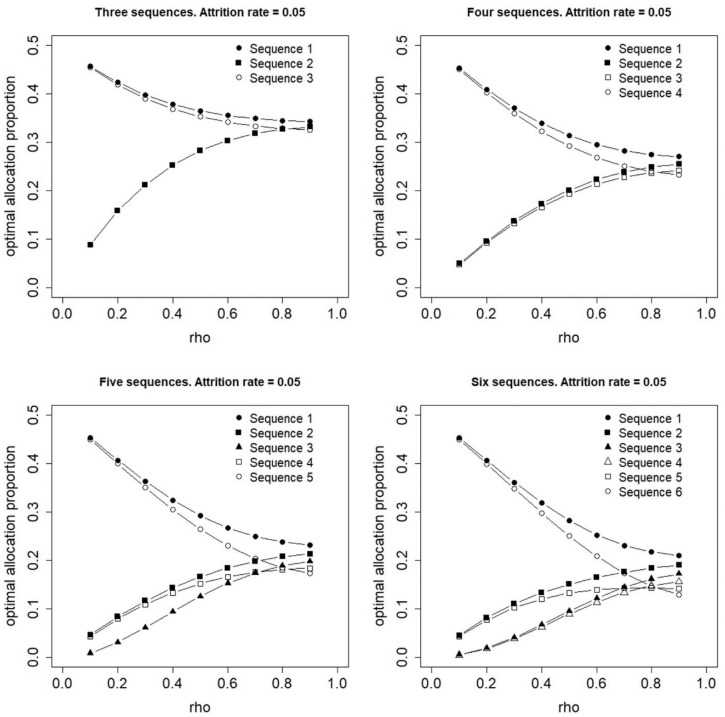
Optimal allocation of individuals to three, four, five or six treatment sequences for a constant attrition rate, r=0.05 , between any two adjacent time points.

**Figure 4.**
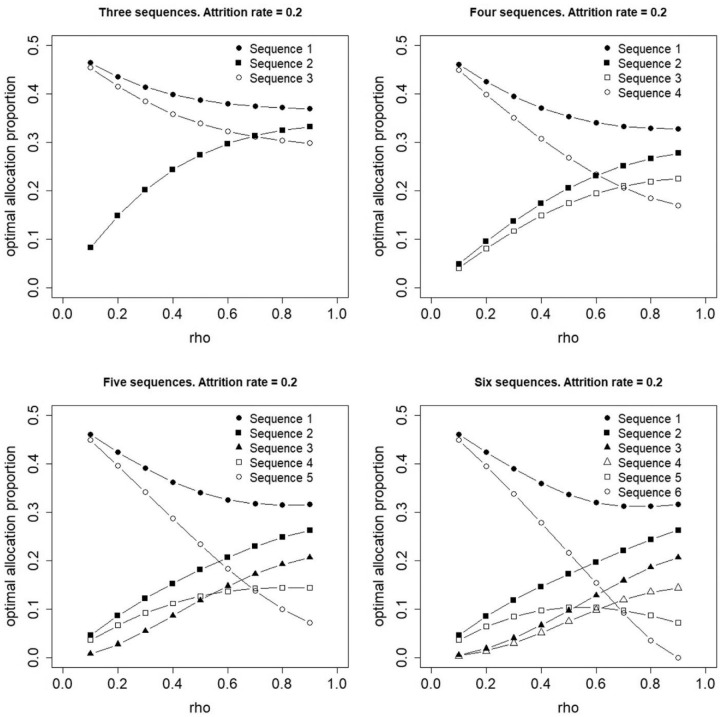
Optimal allocation of individuals to three, four, five or six treatment sequences for a constant attrition rate, r=0.2 , between any two adjacent time points.

Figures 3 and 4 show optimal proportions for a constant attrition rate of r=0.05 and r=0.2 between any two adjacent time points, respectively. The optimal allocation no longer holds its symmetry properties: the first sequence has a higher optimal proportion than the last, the second sequence has a higher optimal proportion than the second last, and so forth. For small values of ρ , sequences closer to the top and bottom edges of the design have higher optimal proportions than those that switch at or near the middle time period(s). This result does not hold for larger values of ρ and if ρ=0.9 , the optimal proportion for a sequence is higher if that sequence has its treatment switch earlier in time. Furthermore, the variability in optimal proportions at ρ=0.9 increases if the attrition rate increases from 0.05 (Figure 3) to 0.2 (Figure 4).

**Figure 5.**
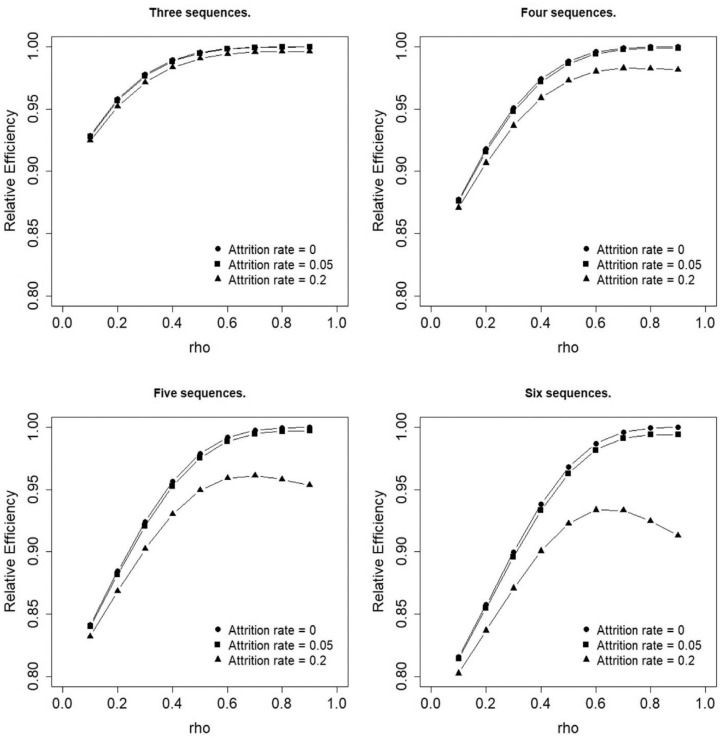
Efficiency of the uniform allocation relative to the optimal allocation.

Figure 5 shows the relative efficiencies of the uniform allocation against the optimal allocations that were presented in Figures 2–4. In all cases, the relative efficiency is at least 0.8 and the loss of efficiency increases with increasing number of sequences. The filled circle lines show the relative efficiencies, in case attrition is absent. In all four panels, larger values are observed for larger values of ρ . In other words: the loss in efficiency when implementing a uniform allocation is smallest if an individual’s observations are highly correlated. The filled square lines show relative efficiencies for attrition rate r=0.05 . As in studies without attrition, there is a monotone increasing relation between the relative efficiency and ρ . The filled triangle lines show the relative efficiencies for attrition rate, r=0.2 . As can be seen, the relation between the relative efficiency and ρ is no longer monotonous: there is a maximum in relative efficiency at ρ=0.6 , after which point increasing correlation reduces the relative efficiency. It should be noted that scenarios with no attrition (i.e. filled circle lines) have the highest relative efficiency while relative efficiencies at higher attrition rates are consistently dominated by those at lower attrition rates.

### Constrained optimal allocation to treatment sequences

The results in Figures 2–4 show that for some values of *ρ *, there is a large variability in the optimal proportions $p_{j}^{*}$ . Some sequences may be assigned a (much) larger proportion of individuals than others. Such an allocation may be impractical as it requires (much) more trained personnel to implement the intervention in one sequence than in another.

Consider as an example a trial with four sequences without attrition (top right panel of Figure 2). Suppose an a priori estimate of the within-person correlation is ρ=0.4 . The optimal allocation is $p^{*}=(p_{1}^{*},p_{2}^{*},p_{3}^{*},p_{4}^{*})=(0.33,0.17,0.17,0.33)$ . A third of the individuals is assigned to sequence 1, only one-sixth to each of sequences 2 and 3 and again a third to sequence 4. This means a relatively large amount of personnel should be recruited and trained to implement the intervention in sequence 1, but only half of them are needed in sequences 2 and 3, and again all of them in sequence 4. If similar personnel numbers are maintained throughout the study, then high workloads at the beginning and end sequences put increased stress on the personnel and resources that may affect intervention implementation and data quality. It could also be that already staffed personnel (like nurses at hospital study sites) are recruited and trained at the beginning of the study, but because half are not needed again until sequence 4 and will likely shift back to their regular duties for a time, there is a risk of them requiring significant retraining. Such types of practical issues can be solved by implementing a constrained optimal allocation.

**Figure 6.**
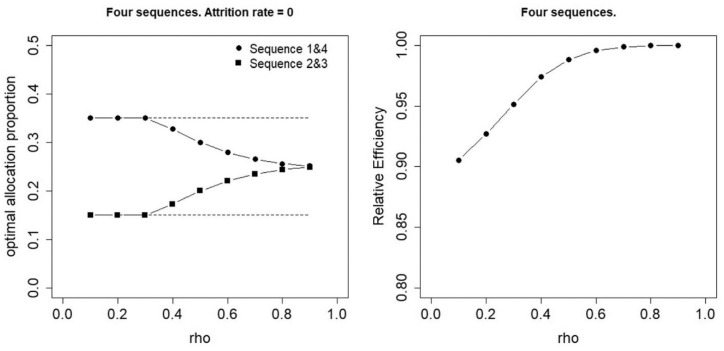
Constrained optimal allocation for four sequences and zero attrition. Left panel: optimal allocation, right panel: efficiency of the uniform allocation relative to the optimal allocation.

Figure 6 shows an example of an optimal allocation with such constraints for a trial with four sequences and absent attrition. The left panel gives the optimal proportions for user-selected constraints on the lower and upper bound $p_{L_{j}}=0.15,\forall j$ and $p_{U_{j}}=0.35,\forall j$ . These two bounds are indicated by the horizontal dashed lines. For ρ≤0.3 , the optimal proportions of sequences 1 and 4 are found on this upper bound and the optimal proportions of sequences 2 and 3 are found on this lower bound. For larger values of ρ , the optimal proportions are as shown in the upper right panel of Figure 2. In other words, for low values of ρ , the optimal proportions are somewhat pulled towards the uniform allocation. As a result of that, the efficiency of the uniform allocation for low values of ρ is somewhat higher when such constraints are used (see right panel in Figure 6) than when they are not (see top right panel in Figure 5).

## Discussion and conclusion

This contribution showed that uniform allocation to treatment sequences is not always the best choice in an individually randomized stepped-wedge design. In studies where attrition is absent, the optimal allocation is almost equal to the uniform allocation in case the repeated measures within an individual are highly correlated. For lower correlations, the optimal proportions may vary much across the sequences. For trials with attrition and a high within-individual correlation, the optimal proportion becomes larger if the sequence has its treatment switch earlier in time. Furthermore, the efficiency of the uniform design decreases when more periods are included in the design, which mirrors the finding for the cohort cluster randomized stepped-wedge designs with a compound symmetry correlation structure.^17^ For all number of sequences J , correlation parameters ρ and attrition rates *r* as studied in this contribution, the efficiency of the uniform allocation is at least 0.8. In other words, for these scenarios, the uniform allocation requires an increase of the sample size of at most 25% to perform as well as the optimal allocation. The (possible) practical limitations of the optimal allocation and the loss of efficiency due to using the uniform allocation should be weighed for any study at hand before a decision is made about the most suitable allocation of individuals to treatment sequences.

R syntax to calculate the optimal allocation to sequences can be found on my Github page. The optimal allocation is locally optimal, meaning it depends on the correlation parameter ρ and attrition rate *r*. A priori values of these two parameters may be found in the literature or obtained from expert knowledge. A sensitivity analysis may be performed to study how the optimal allocation changes if other plausible values of these parameters are used. Alternatively, one may derive a more formal robust design, such as a maximin design. To facilitate researchers of future stepped-wedge designs, I advocate estimates of the correlation parameter and attrition rate are clearly reported in the scientific literature.

The stepped-wedge design is a longitudinal design and is therefore subject to attrition. This contribution studied optimal allocation for attrition that was constant across time and future research may focus on attrition rates that change over time. In my previous research on longitudinal studies, I used the Weibull survival function, which allows for monotonically increasing or decreasing attrition over time.^23,24^ It would be of interest to study how the optimal allocation of an individually stepped-wedge design behaves under such attrition. This contribution also assumed that attrition is constant across individuals. Future research may focus on optimal allocations when attrition depends on, for instance, the treatment sequence. For instance, attrition may be higher for individuals in the less interesting control condition than those in the intervention condition. It may then be expected that more individuals are allocated to those sequences that have their treatment switch earlier in time.

Missing data are often considered a burden: they make the design incomplete rather than complete and hence result in a loss of efficiency. However, incomplete designs may be considered a priori if the aim is to minimize the number of measurements rather than the number of individuals. In the social science literature, such designs are known as planned missing data designs.^25^ An example is the so-called dog-leg design^26^ and it may be interesting to study optimal allocations to treatment sequences for such a design.

Another design for which it may be interesting to study the optimal allocation is the so-called hybrid design or sandwich stepped-wedge design.^16,27,28^ This is a combination of the multi-period parallel-group design and the stepped-wedge design. For cluster randomized trials with large samples, this design has been shown to be more efficient than the stepped-wedge design. It would be interesting to see if this result translates to the individually randomized stepped-wedge design and how the optimal allocation is affected by attrition.

It should finally be mentioned that this contribution assumes a sustained effect of treatment. This assumption does not always hold in practice: the effect of treatment may be pronounced shortly after the treatment switch, but then stabilize or even decrease later in time if the initial treatment benefits are washed out over time. This may be taken into account in the statistical model by allowing for differential treatment effects after the treatment switch. The allocation that is optimal for one such treatment effect may not be so for another. To derive an ‘overall’ optimal allocation, one may derive a so-called $D_{S}$ -optimal design. Such an optimal design minimizes the confidence ellipsoid of a subset of all model parameters, where in this case the subset consists of all treatment effects.^29,30^ Another approach may be to minimize the weighted sum of variances of these treatment effects in a compound optimal design,^31^ where the weights represent the relative importance of the treatment effects.

To my knowledge, this is one of the first studies on the efficient design of the individually randomized stepped-wedge design. I hope the results in this article will help medical scientists to design their trial in an efficient way. I also hope this contribution will increase methodological interest in the individually randomized stepped wedge, so that more guidelines for its design and analysis will become available in the near future.
